# Transforming healthcare documentation: harnessing the potential of AI to generate discharge summaries

**DOI:** 10.3399/BJGPO.2023.0116

**Published:** 2024-02-07

**Authors:** Reece Alexander James Clough, William Anthony Sparkes, Oliver Thomas Clough, Joshua Thomas Sykes, Alexander Thomas Steventon, Kate King

**Affiliations:** 1 Institute of Naval Medicine, Gosport, UK; 2 Academic Department of Military General Practice, Research & Clinical Innovation, Defence Medical Services, ICT Centre,, Birmingham, UK

**Keywords:** artificial intelligence, ChatGPT, discharge summary, general practice, feasibility studies

## Abstract

**Background:**

Hospital discharge summaries play an essential role in informing GPs of recent admissions to ensure excellent continuity of care and prevent adverse events; however, they are notoriously poorly written, time-consuming, and can result in delayed discharge.

**Aim:**

To evaluate the potential of artificial intelligence (AI) to produce high-quality discharge summaries equivalent to the level of a doctor who has completed the UK Foundation Programme.

**Design & setting:**

Feasibility study using 25 mock patient vignettes.

**Method:**

Twenty-five mock patient vignettes were written by the authors. Five junior doctors wrote discharge summaries from the case vignettes (five each). The same case vignettes were input into ChatGPT. In total, 50 discharge summaries were generated; 25 by Al and 25 by junior doctors. Quality and suitability were determined through both independent GP evaluators and adherence to a minimum dataset.

**Results:**

Of the 25 AI-written discharge summaries 100% were deemed by GPs to be of an acceptable quality compared with 92% of the junior doctor summaries. They both showed a mean compliance of 97% with the minimum dataset. In addition, the ability of GPs to determine if the summary was written by ChatGPT was poor, with only a 60% accuracy of detection. Similarly, when run through an AI-detection tool all were recognised as being very unlikely to be written by AI.

**Conclusion:**

AI has proven to produce discharge summaries of equivalent quality to a junior doctor who has completed the UK Foundation Programme; however, larger studies with real-world patient data with NHS-approved AI tools will need to be conducted.

## How this fits in

Primary care relies on good-quality discharge summaries to ensure good-quality continuity of care. Junior doctors are currently overstretched, and AI integrated into electronic patient record (EPR) software could relieve some of the burden through the generation of discharge summaries using coded entries. This feasibility study showed that AI can produce discharge summaries at the same level as a junior doctor, in less time, with minimal human input.

## Introduction

We are on the cusp of an artificial intelligence (AI) revolution, which is rapidly evolving and will undoubtedly impact enterprises worldwide, with health care being no exception. The World Health Organization produced its first global report on AI in health in 2021, which emphasised the importance of ethics and human rights at the forefront of its design, deployment, and use.^1^ The NHS has voiced its commitment to exploring AI, with the *NHS Long Term Plan* stating that AI needs to be embraced providing it is used correctly.^2^ The plan recognises that, in the coming years, the quality of AI will improve, many tasks will be automated, and staff released to focus on the complex and empathetic human interactions that technology will never master.^2^ This is reflected by the NHS AI Lab investing £123 million in 86 innovations since its inception in 2019.^3^

One tool attracting public interest is ChatGPT by OpenAI L.L.C, 2022.^4^ ChatGPT is a type of natural language processing model known as large language model (LLM). It is trained on a massive amount of text data with the aim of generating human-like responses to a given input.^5^ From a simple query it can perform a multitude of tasks ranging from meal plans to complex coding problems. The use of LLMs in healthcare documentation is increasingly being explored.

Discharge summaries are an essential communication tool to give GPs insight into a patient’s hospitalisation, to ensure effective continuity of care, and prevent adverse events. Electronic discharge summaries are available, but these are based on fixed coding of information and of limited value to GPs.^6^ Usually, however, discharge summaries are written by the most junior doctors on the team. Training in this task is poor and, with variable time available for the task, they inherently vary in quality.^7,8^ They often lack important information such as test results, treatments received, medications, and follow-up plans.^9^ Medication information errors are a particular patient safety risk, with transcription errors found in both manual and electronic discharge summaries.^10^ Abbreviations are often misunderstood with different meanings attributed by authors and recipients.^11^ A systems approach to enhancing communication between hospital and GPs can improve information flow, and ensure accurate and timely transmission of discharge summaries.^12^

Frameworks aiming to improve the quality of discharge summaries are available,^13,14^ but there is no nationally accepted model currently in place. Even with a standard, adherence can be limited with one audit finding mean adherence of 71.7% in 2444 discharge summaries.^15^ Templates built-in to electronic patient records may improve adherence,^15^ but an efficient and streamlined process is important, and it is here that AI-generated summaries could be the key to improving communication during hospital discharge. The standardisation and clarity of information in AI-generated summaries could address all the existing issues.

ChatGPT has the potential to generate hospital discharge summaries.^16^ LLMs are exceptionally good at using protocols, in a repeatable, timely, and cost-effective manner. Utilisation of such tools may help relieve a large burden on an already stretched junior doctor workforce, thereby increasing the amount of time they have in the working day for direct patient care, professional development, and ensuring patients are discharged in a timely manner with quality discharge documentation.

Although previously shown to work, ChatGPT's ability to produce more personalised high-quality summaries has not yet been assessed. This study aimed to look at the ability of ChatGPT to generate discharge summaries for a variety of presentations, and assess the quality of its work when compared with that of a doctor who has completed the UK Foundation Programme.

## Method

**Figure 1.**
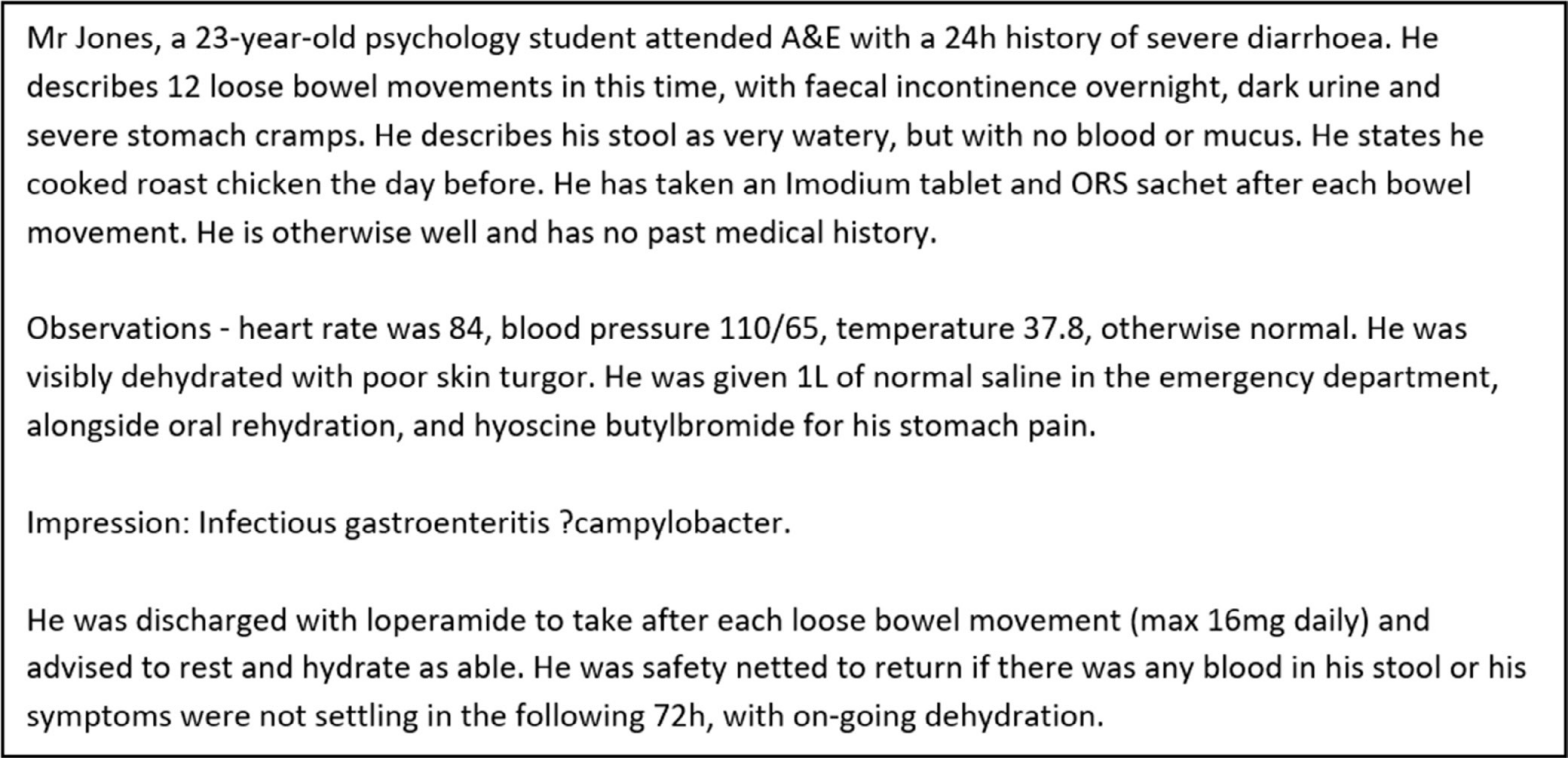
Example of a mock patient vignette

A range of 25 case vignettes were written by the authors. Each contained pertinent information such as patient demographics, diagnosis and reason for admission, past medical history, investigations conducted, treatments received, and any discharge recommendations. The cases covered a range of specialties and were loosely based on scenarios encountered by the authors during their Foundation Programme. An example case vignette is shown in Figure 1. No actual patient data were used in the study.

**Figure 2.**
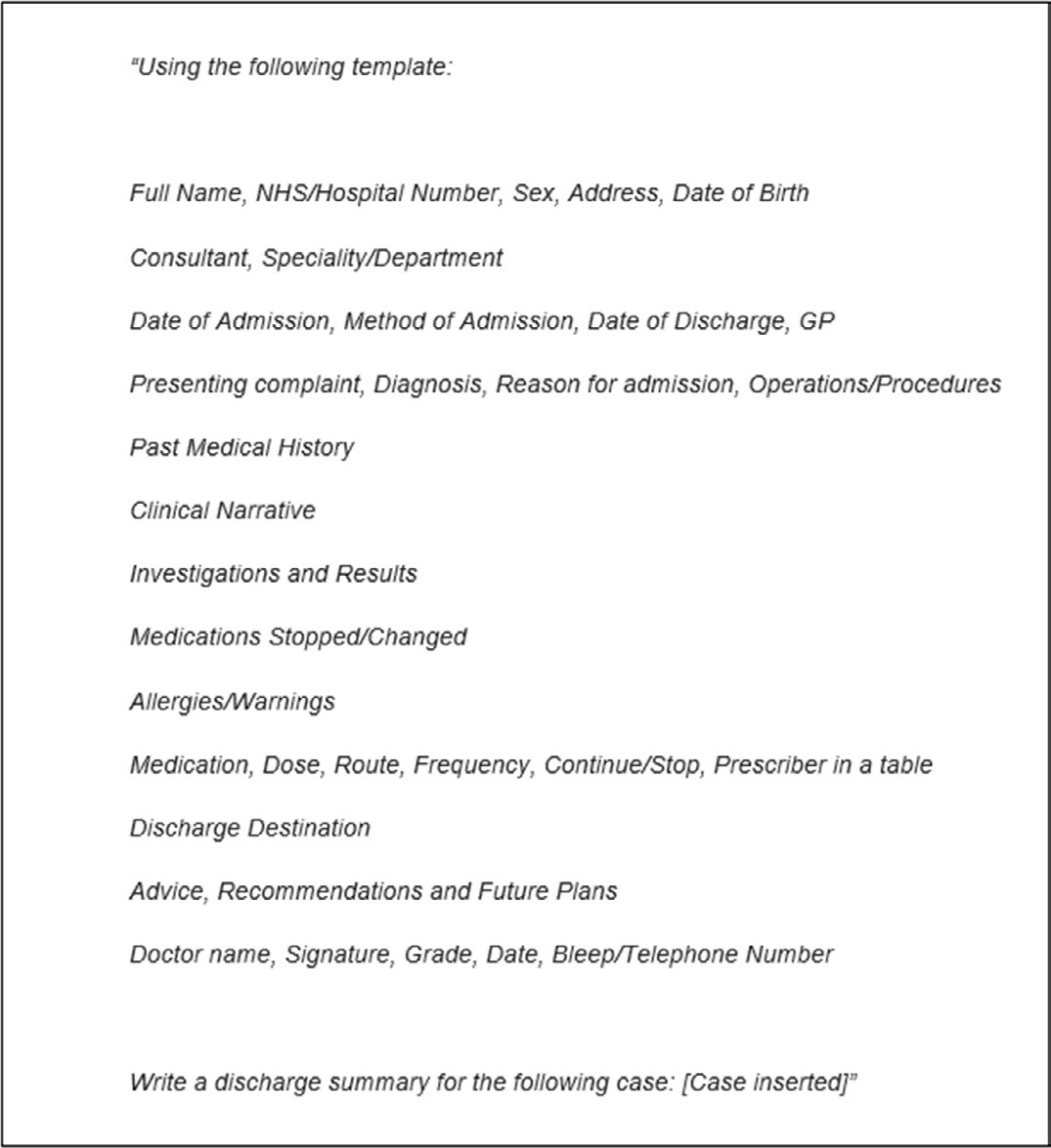
Initial prompt used in ChatGPT to generate discharge summary

**Figure 3.**
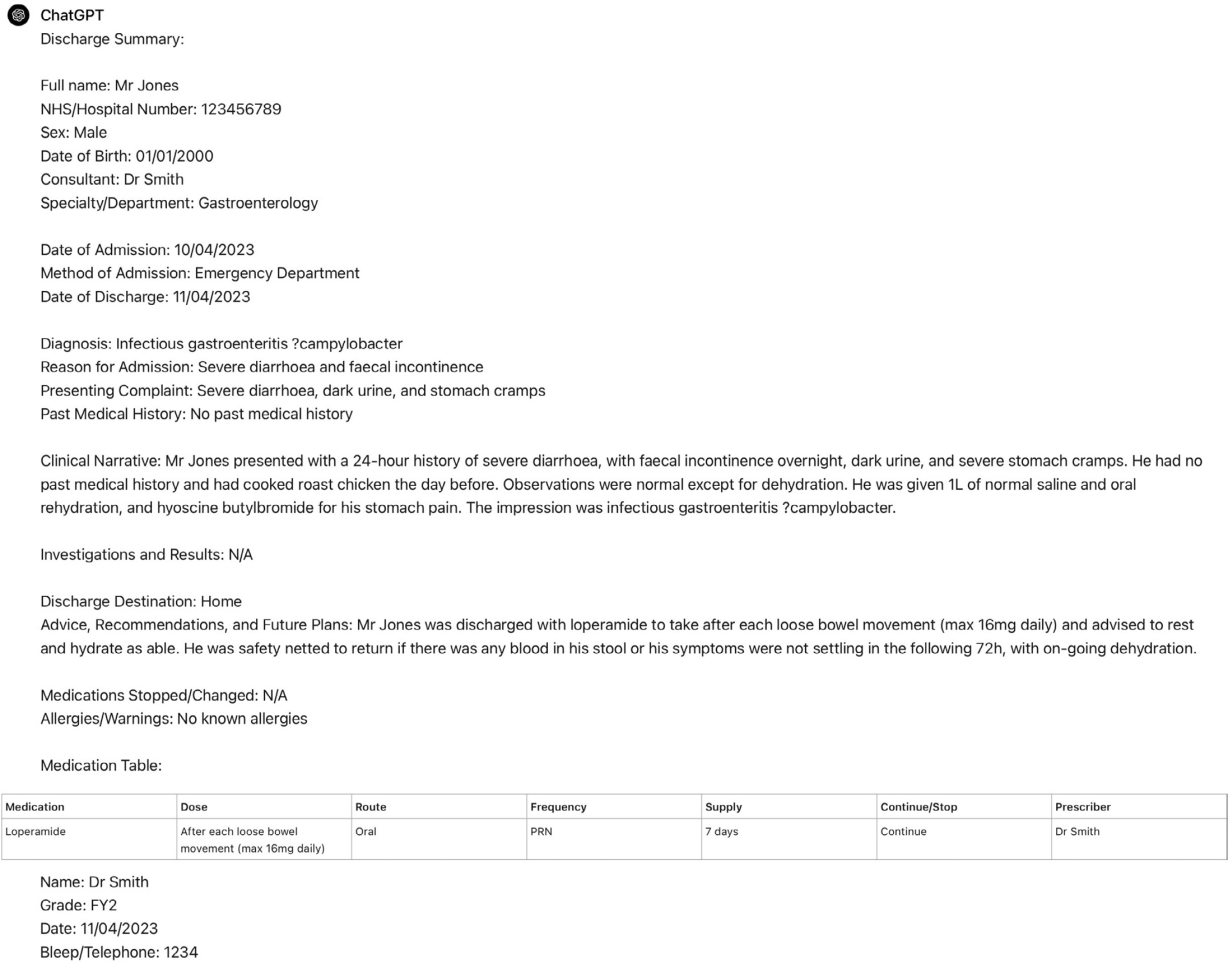
Example of a ChatGPT-produced discharge summary

Five junior doctors of comparable experience (all having completed the UK Foundation Programme, but not progressed significantly through specialty training) then wrote discharge summaries from the case vignettes (five each). The same 25 vignettes were input into ChatGPT using the same initial prompt that was devised by our team based on our experiences of what a discharge summary should include (Figure 2). No specific training was given to the AI and the only follow-on prompts used were to address formatting; for example, placing medications into a table rather than a list. An example ChatGPT summary is shown at Figure 3. A total of 50 discharge summaries were produced; 25 AI-written summaries and 25 doctor-written summaries.

Five GPs were recruited through word of mouth. They were asked to be part of a study looking at whether AI could generate a discharge summary. The lead author assigned each GP 10 summaries to review, ensuring a mix of doctor and ChatGPT written summaries. The reviewing GPs were blinded to the origin of the discharge summaries they reviewed. They were then asked to answer the following for each summary:

1. Do you think this discharge summary was written by AI?
2. Would you be happy to receive this discharge summary for a patient in your practice?

**Figure 4.**
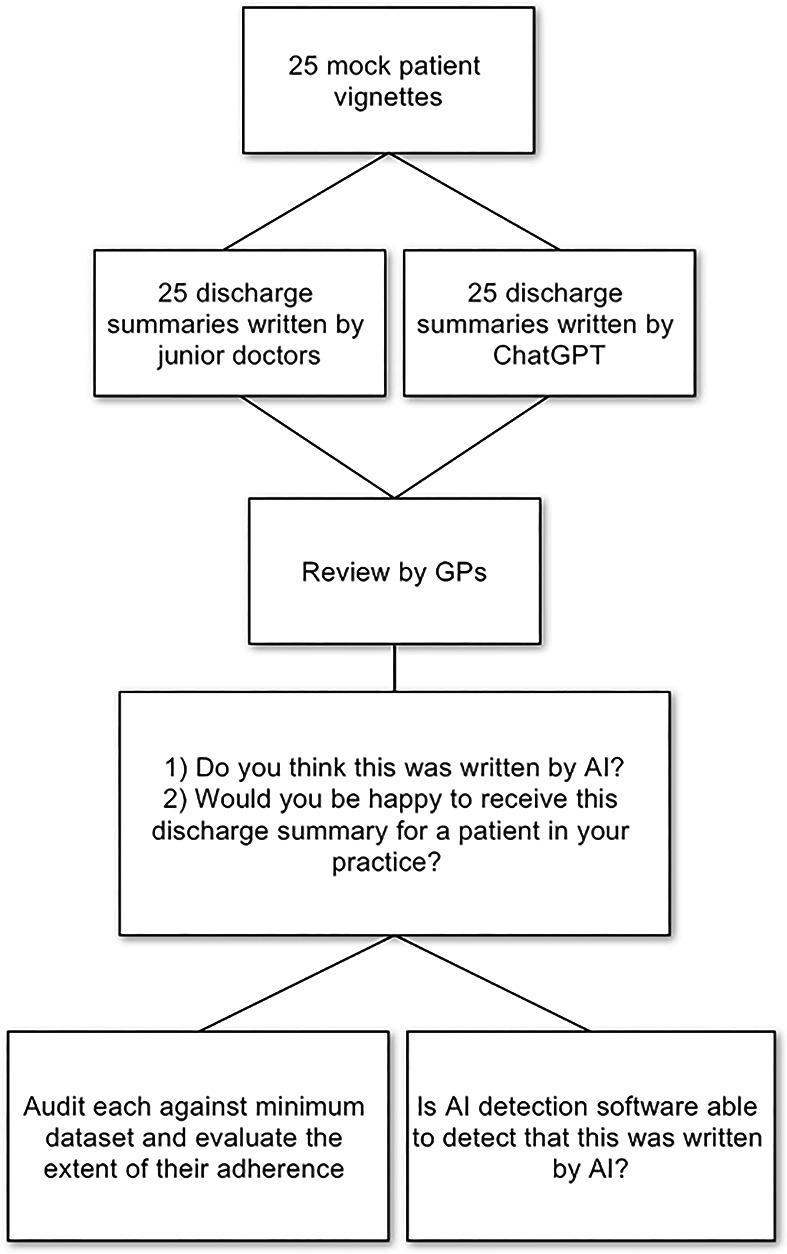
Summarised study methodology. AI = artificial intelligence

Each discharge summary was then evaluated using the National Prescribing Centre (NPC) minimum dataset.^13^ This consisted of 19 equally weighted criteria and allowed an adherence score to be calculated to compare both groups. This is shown in Table 1. Finally, to determine if the ChatGPT-generated discharge summaries could be detected as written by AI, the summaries were processed through the OpenAI Text Classifier,^17^ which labelled them as either very unlikely, unlikely, unclear if it is, possibly, or likely AI-generated. The methodology is summarised in Figure 4.

**Table 1.** Discharge summary minimum dataset

Demographics and admission details
Correct patient name
Correct date of birth
Consultant name
Ward
Date of admission
Date of discharge
Presenting
Complete past medical history and comorbidities
Complete medical history
Known allergic or hypersensitivities
Discharge summary is legible
**Medication information**
All doses
All frequencies
All routes of administration
All formulations
Therapy duration when a medication was initiated by hospital team where this was appropriate (for example, antibiotics, short-course corticosteroids, hypnotics)
**Therapy changes information**
All medicines initiated with reason(s)
All medicines discontinued with reason(s)
All medicines changed with reason(s)

### Statistical analysis

A Fisher’s exact test was used to assess if there was a statistically significant difference in GP acceptance rate between the AI and doctor-generated summaries. To determine the ability of GPs to detect if a discharge summary was AI-generated, we looked at the number of true positives, false positives, true negatives, and false negatives taking the correct identification of an AI-written discharge summary as a true positive. Accuracy, sensitivity, specificity, and positive predictive values (PPV) were determined for each. The minimum quality dataset score adherence percentage was calculated for each summary and a Mann–Whitney U test was performed to compare adherence with the dataset between the two groups.

## Results

GPs stated that they would accept 48 of the 50 discharge summaries in their practice; all of the ChatGPT written summaries (100%) and 23 out of 25 of the junior doctor written summaries (92%). This gave a mean acceptance of 1.00 for the ChatGPT group and 0.92 for the junior doctor group (*P =* 0.15).

GPs correctly identified 13 of the AI summaries and falsely believed that eight doctor-written discharge summaries were written by AI. This gave an overall accuracy of detection of 0.6, with a sensitivity of 0.52, specificity of 0.68, and a PPV of 0.62.

**Figure 5.**
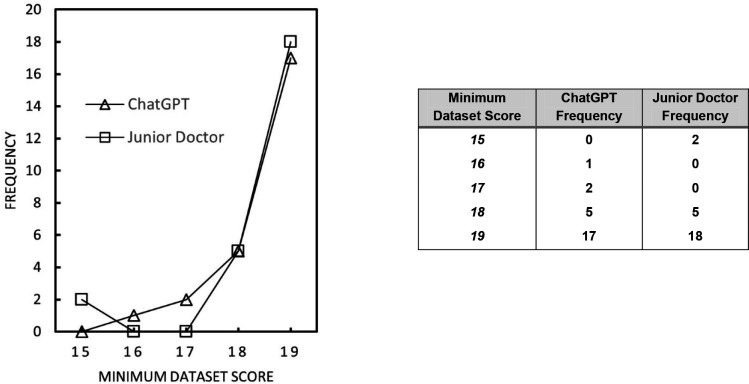
Minimum dataset score achieved for ChatGPT and junior doctor-generated discharge summaries

On analysing the adherence of each group to the minimum dataset, it was found that all discharge summaries scored ≥15, with both groups showing a median score of 19 and a 97% mean adherence. Results are summarised in Figure 5. There was no significant difference between the minimun dataset scores of the AI group and the doctor group, *z* = –0.28, *P* = 0.78.

When analysed by the OpenAI AI Text Classifier, it was found that all AI-produced discharge summaries were classified as 'very unlikely' to be generated by AI, suggesting that the GPs were better at identifying AI than the AI classifier.

## Discussion

### Summary

This study has shown that ChatGPT has the potential to produce discharge summaries of sufficient quality to satisfy GPs and which are equivalent to those written by a junior doctor. The ChatGPT summaries were not readily identified as being AI written and there was no significant difference in their quality compared with the doctor-written summaries.

### Strengths and limitations

The major strength of this study is that it was able to meet the primary aim and show, through both independent GP evaluation and adherence to minimum dataset criteria, that AI can produce a hospital discharge summary at the same quality as a junior doctor.

Study limitations relate to the small sample size, single-GP rating of each summary, and the requirement to use mock patient vignettes, which lack some of the complexities of real-life patient records, which may include multiple entries and previous admissions. As this was a feasibility study to determine whether AI could produce satisfactory discharge summaries, the small sample size is considered a minor limitation, although it is accepted that a larger sample size may have elicited a significant difference between the GP satisfaction with the summaries produced by the groups. Each discharge summary was reviewed by only one GP; this may have led to variations in inter-rater reliability, underpinned by individuals’ preconceived notions, opinions, or attitudes about AI. While this was mitigated against (through blinding the GPs to the case authors) future studies should ensure each summary is evaluated by multiple GPs.

The use of case vignettes was necessary as ChatGPT cannot access medical records. For this result to be replicated in the real world, ChatGPT would need to be able to access clinical notes and identify the relevant information from the data generated at each admission.

The study showed that ChatGPT can produce effective discharge summaries from a case vignette when given a pre-determined template to generate the correct format. Limitations associated with access to patient notes and the ChatGPT system itself meant that the input needed to be in the format of a vignette. However, it has been shown that AI can already accurately extract medication-related information from clinical documents,^18^ which can be leveraged to supplement discharge summaries. Adapting the programming of current AI systems to capture and distill the relevant clinical information from notes relating to a hospital admission is still a big leap, but at the rate of technological advancement and increasing digitalisation of hospital notes, it seems like it is only a matter of time.

### Comparison with existing literature

This study has added to the increasing evidence of AI being of benefit in the clinical environment. Several studies have explored the use of AI in generating patient clinic letters, including discharge summaries.^16,19,20^ The studies are generally positive and, like this one, suggest that AI has a future role, but there are still logistical or even ethical issues that need to be overcome before widescale integration of AI into clinical communication processes is possible.

Ali *et al* showed that ChatGPT could effectively generate clinic letters and showcased the efficiency of the process,^19^ but, again, required a single-source data input akin to the case vignettes. When examining whether AI can generate discharge summaries from inpatient records, it was shown that the origins of information contained in a discharge summary is varied and 39% of the information comes from the inpatient record, with 11% deriving from the physician’s memory.^20^ This raises concerns with the feasibility of AI involvement in clinical communication, which aligns with the data governance issues raised by Patel and Lam.^16^ They discussed the potential of using ChatGPT to generate discharge summaries and, while they recognised the benefits of automation, standardisation, and efficiency, they also highlighted that errors are frequent, and a minor error could have catastrophic consequences in the clinical environment.^16^ They, like Ando *et al*, have recommended that any future AI involvement will always need human oversight to minimise patient safety risks as well as providing more personalised information where needed.^16,20^

### Implications for research and practice

AI has the potential to change how health care is delivered. However, to bring about change, trust in AI by major stakeholders is required. It is encouraging that the *NHS Long Term Plan* recognise the benefits AI could offer,^2^ but the perceptions of healthcare professionals also needs to be taken into consideration. In addition, the cost of developing and integrating AI into existing systems can be expensive and challenging.

AI relies on vast amounts of data to be trained and could result in incomplete or inaccurate information or reinforce existing biases in health care. Previous AI algorithms, for example, have been shown to be more likely to interpret images of skin lesions as malignant if they contained a ruler.^21^ The AI had inadvertently 'learnt' that the presence of a ruler meant the lesion was malignant. These biases can exist unless the AI is trained to ignore such information.

There is no fear that AI will entirely replace healthcare professionals^22^ and the authors envisage AI supplementing the work of junior doctors rather than replacing them. Currently, AI has limited ability to infer, from the text it uses, reasons why clinical decisions were made. High-quality discharge summaries need to not only convey what happened but also provide the GP with clinical reasoning. An AI-human hybrid process would better ensure that the reasoning behind clinical decisions is transparent.

If AI was to be implemented directly into electronic patient record (EPR) software, it would rely on the coded entries to find the relevant information. This has the same limitations as existing electronic discharge summaries; its quality is only as good as the coding of information. The advantage of AI comes from its potential to extract information from natural language data; the prose rather than the discrete coded information. This requires the AI tool to have access to digital data, and currently only around 80% of NHS trusts have EPR systems.^23^

AI can be trained to consistently produce output to a standard. Automation brings less variation than is seen with human documentation, meaning, once agreed minimum requirements are determined, AI can collate this information much faster. Furthermore, AI can learn from its mistakes and ensure any flagged missed information is included. However, it does create the potential for discharge summaries to be too generic. This may fail to capture and effectively communicate the individual nuances of a patient’s treatment, and provide important patient-specific information, which is not part of a standard dataset. This depersonalisation may leave patients feeling that information important to them is not included, leading to decreased patient satisfaction and engagement in their own care.

One interesting advantage of AI, which has not yet been explored in the literature, is the ability to simplify written information. Open communication with patients underpins the digitisation of NHS health care. The *NHS Long Term Plan* aims for all patients to have access to their clinical notes by 2023–2024.^2^ As of November 2023 21.8 million patients have full prospective health record access with 72% of GP practices (4557) providing this as access by default.^24^ However, clinicians share a common language; one that is not always shared by or accessible to patients. Medical language is beneficial when clinicians communicate, but for patients it can be jargonistic and impenetrable. Patients sometimes turn to their GPs for interpretation services when they are sent a copy of a clinic letter, adding to the already overburdened GP appointment load. Exploiting AI’s ability to simplify information could result in the production of lay summaries and patient education materials following clinical contacts, which could enhance their understanding of their hospitalisation and ongoing management.

In conclusion, AI has the potential to transform healthcare documentation by automating the generation of discharge summaries. This study has added to the evidence that there is a promising future for specialised AI systems supporting healthcare communication. The GP is integral to health care in the UK, and when AI is incorporated into standard working procedures, it is vital that GPs are involved in defining the information content for communication. Additionally, AI used to improve patient-facing communication should be explored. Further investigation with NHS-approved tools and real-world patient data is required before AI can be adopted but, with rates of technological expansion doubling every 2 years,^25^ it is likely that AI support will be available to doctors in the not too distant future.

## Data

The dataset relied on in this article is available from the corresponding author on reasonable request.
